# Supplementary material for: Deletion of wfs1 Impairs Oligodendrocyte Precursor Cells Dorsal Distribution and Myelination Through the wfs1-hmgcs1 Axis in Zebrafish
Source: Biology (Basel). 2026 Aug 16;15(16):1402. doi: 10.3390/biology15161402 (PMC13510151; doi:10.3390/biology15161402)
Supplement: Supplementary file 1 [file biology-15-01402-s001.zip › File S1-Transcriptome sequencing data and differentially expressed genes (DEGs) in wfs1-- zebrafish at 5 dpf/2.KEGG/wfs1KOvsWT/ALL/src/dre04150.html]

dre04150


- dre:245702

- Up regulated genes

245702(1.27755220735994)

- Down regulated genes

245702\_1(-1.45433245048201)

- dre:100000252

- Up regulated genes

100000252\_3(1.94434154854557) 100000252(1.09163460881879)

- Down regulated genes

100000252\_1(-1.7718817168434)

- dre:100330617

- Up regulated genes

100330617\_1(1.54741913113035)

- dre:799099

- Up regulated genes

799099\_1(1.05373800076525)

- dre:436718

- Up regulated genes

436718(1.34229427553561)

- Down regulated genes

436718\_1(-6.85571323128554)

- dre:100006675

- Up regulated genes

100006675(1.63301233543285)
- dre:497384

- Down regulated genes

497384\_1(-2.05267237809407)

- dre:324140

- Up regulated genes

324140\_1(4.93450118149538)

- Down regulated genes

324140(-5.90052475401947)

- dre:573492

- Up regulated genes

573492\_1(2.04323918457063)

- Down regulated genes

573492(-2.61487825195395)

- dre:100009657

- Up regulated genes

100009657\_1(1.89754653566672)

- dre:79380

- Up regulated genes

79380(9.11141682868863)

- Down regulated genes

79380\_1(-1.05168581152728)

- dre:797250

- Down regulated genes

797250(-1.06740189529136)

- dre:796322

- Up regulated genes

796322(2.03500950739103)
- dre:790938

- Down regulated genes

790938(-4.50740250004449)

- dre:563708

- Down regulated genes

563708(-3.59233336704936)

- dre:359840

- Up regulated genes

359840(3.81608997342262)

- Down regulated genes

359840\_1(-1.77685900658053)
- dre:359839

- Up regulated genes

359839(1.49784899389719)

- dre:100333504

- Down regulated genes

100333504\_1(-6.61871930566475)

- dre:336619

- Down regulated genes

336619(-2.2262247407159)
- dre:553214

- Up regulated genes

553214\_1(2.81125103603555)

- dre:792689

- Down regulated genes

792689\_1(-1.78857482493481)

- dre:555584

- Up regulated genes

555584\_1(1.87118961020207)

- Down regulated genes

555584(-4.00454883626176)

- dre:792689

- Down regulated genes

792689\_1(-1.78857482493481)

- dre:556087

- Up regulated genes

556087(1.25789886083115)

- Down regulated genes

556087\_1(-6.16688081226611)
- dre:791993

- Down regulated genes

791993(-3.69340367298196)
- dre:30127

- Down regulated genes

30127\_1(-1.48241226857528)

- dre:58072

- Up regulated genes

58072\_1(2.20804115837263)

- Down regulated genes

58072(-6.00118378322126)
- dre:30369

- Down regulated genes

30369\_1(-1.70732701220414)

Close
